# Hybrid quantum/classical docking of covalent and non-covalent ligands with Attracting Cavities

**DOI:** 10.1038/s41598-025-24614-3

**Published:** 2025-11-26

**Authors:** Mathilde Goullieux, Vincent Zoete, Ute F. Röhrig

**Affiliations:** 1 Molecular Modeling Group, SIB Swiss Institute of Bioinformatics, CH-1015 Lausanne, Switzerland; 2 Department of Oncology, Ludwig Institute for Cancer Research Lausanne Branch, Lausanne University (UNIL), CH-1066 Epalinges, Switzerland; 3 Present Address: Center For Electron Laser Science CFEL, Deutsches Elektronen-Synchrotron DESY, Notkestr. 85, 22607 Hamburg, Germany

**Keywords:** Docking, QM/MM calculations, Covalent ligands, Hemoproteins, Drug discovery, Structure-based drug design

## Abstract

Ligand–protein docking aims to predict how a ligand binds to a biological macromolecule and is a fundamental technique in structure-based computer-aided drug design. However, accurately modeling covalent binding, metal coordination, and polarization effects remains challenging for classical docking algorithms. Here, we present an extension of our Attracting Cavities docking algorithm that enables hybrid quantum mechanical/molecular mechanical (QM/MM) calculations at various levels of theory. To evaluate its performance, we benchmarked the method on three diverse datasets covering non-covalent drug–target complexes, covalent complexes, and hemoprotein complexes, using both semi-empirical and density functional theory approaches. The results demonstrate that QM/MM docking is especially advantageous for metal-binding complexes, where the fast semi-empirical PM7 method yields a significant improvement over classical docking. When describing the active site residues at the density functional theory level, dispersion corrections are crucial for meaningful energies. Overall, the QM/MM method outperforms the classical approach for metalloproteins, performs comparably for covalent complexes, and shows slightly lower success rates for non-covalent complexes.

**Supplementary Information:**

The online version contains supplementary material available at 10.1038/s41598-025-24614-3.

## Introduction

Computer-aided drug design (CADD) is a standard tool in modern drug discovery. Molecular docking plays a central role in CADD by predicting how a ligand binds to a biological macromolecule. Docking algorithms face several key challenges, including accounting for receptor flexibility, solvation effects, polarization, and metal or covalent binding^1^. The latter two issues are inherently quantum chemical in nature and can be effectively addressed using hybrid quantum mechanical/molecular mechanical (QM/MM) methods. In biological contexts, such challenges – traditionally handled with classical force fields (FF) – have been investigated with QM/MM approaches^2^, particularly in studies of enzymatic reactions^3–5^, metalloproteins^6^, and protein photochemistry^7^.

Half of todays’ known proteins contain a metallic binding site, responsible for many processes, such as electron transfer^8^, gas sensing and transport^9^, reaction catalysis^10^and others. Due to the number of reactions in which they take part, metalloproteins are involved in many diseases such as in cancer, bacterial infections, or neurodegenerative disorders. Therefore, it is of utmost importance to develop drugs which efficiently target or inhibit them^11,12^. Designing inhibitors for these proteins with CADD requires an accurate representation of their binding interactions. For many years, specific FF able to model metalloproteins have been developed^13^. For instance, GOLD^14^ and FlexX^15^ include parameterized knowledge-based scoring functions, able to treat various metal ions in docking. Autodock4Zn^16^ and MpSDock^17^ include specifically refined scoring functions for docking to zinc metalloproteins, which represent an important sub-family of such systems.

In a previous study, our group demonstrated that use of QM calculations in docking is advantageous^18,19^. We developed a hybrid on-the-fly QM/MM docking algorithm based on our classical docking algorithm Attracting Cavities (AC)^20,21^ and the semiempirical (SE) self-consistent charge density functional tight-binding (SCC-DFTB) QM/MM interface^22^of the CHARMM program^23^. We benchmarked the performance of the code on the Astex Diverse set^24^, a zinc metalloprotein dataset^18,25^, and a hemeprotein dataset^19,26^. Redocking performance of the on-the-fly QM/MM docking method preserved the high accuracy of classical scores on the Astex Diverse set and yielded significant improvements on both sets of metalloproteins at low computational cost. However, due to the lack of SCC-DFTB parameters, complexes with iron-sulfur or iron-halogen interactions had to be excluded from the dataset, eliminating all hemoproteins with an iron-cysteine bond. Therefore, we pursued the development of a QM/MM docking code with the aim to improve the QM description and to expand its applicability to a wider range of systems, including covalent complexes.

Covalent drugs are nowadays commonly used in medicinal chemistry due to their many advantages^27^. As a result, accurately describing and predicting their interactions with biological targets is crucial, and many docking algorithms provide an option for covalent docking, for example AutoDock (AD)^28,29^, Glide (CovDock)^30,31^, FITTED^32–35^, ICM-Pro^36,37^, and GOLD^38–40^. Other programs were specifically developed for covalent docking, such as CovalentDock (built on AutoDock)^41^, Cov_DOX^42^, Covalent CDOCKER^43^, WIDOCK^44^, and HCovDock^45^. To simulate the two-step process of covalent ligand binding, we previously developed a covalent docking approach in Attracting Cavities (AC), where the ligand binds to the receptor through non-bonded interactions before a chemical reaction forms the covalent bond^46^. The method demonstrated promising results on a set of 304 complexes, reaching a success rate of 78% in reproducing the native pose with a RMSD $\le$ 2 Å, outperforming the popular docking codes AD^29^and GOLD^40^.

Classical docking codes face the challenge of accurately estimating the energy of the covalent bond. Therefore, several research groups are developing approaches that incorporate quantum mechanical (QM) calculations to improve the docking of covalent ligands. For instance, WIDOCK includes parameters accounting for ligand reactivity towards cysteine residues, derived from experimental reaction kinetics or from computed quantum chemical reaction barriers^44^. As a further example, Cov_DOX is a multi-scale QM docking algorithm with three levels of potential energy refinement, coupling classical FF, PM7, and DFT calculations^42^. The authors report a re-docking success rate of 58% at the PM7 level and of 81% at the DFT level for a benchmark set^47,48^of 405 covalent ligand-protein complexes, suggesting that QM methods are favorable for refining the description of covalent systems^42^. However, we showed that only 64% of complexes in this benchmark set pass basic quality filters such as a good resolution ($\le$2.5 Å), complete atom coordinates, a unique ligand conformation, and a well-ordered ligand (average ligand B-factor $\le$80 Å$^2$, see Table 1in Ref^46^. for a detailed list of criteria). In our hands, the quality of experimental data is paramount to achieving high re-docking success rates^21,46^. Unfortunately, the Cov_DOX webserver is not accessible, making it impossible to independently assess its speed and performance.

In the present study, we developed a QM/MM algorithm in AC, relying on the QM/MM interface of CHARMM^23^with the Gaussian quantum mechanics code^49^, giving access to a wide range of *ab initio*, density functional theory (DFT), and semi-empirical methodologies and basis sets. The algorithm is able to treat many types of ligands and receptors, including RNA, DNA, metalloproteins, and covalently bound ligands. We benchmarked this algorithm on three diverse sets of ligand–receptor complexes, (1) the Astex Diverse set of 85 curated complexes of non-covalent drug-like ligands^24^, (2) the high-quality and diverse CSKDE56 set of 56 covalent complexes^46^, and (3) a new high-quality set of 70 heme-binding complexes (HemeC70). We investigate different parameters influencing the docking results in detail and show that the QM/MM approach outperforms the classical one for metalloproteins, reaches similar success rates for covalent complexes, and slightly lower ones for non-covalent complexes.

## Methods

### Benchmark sets

In this study, we used three diverse sets of ligand–protein complexes to benchmark our QM/MM docking algorithm. The Astex set of 85 manually curated complexes of diverse and drug-like protein–ligand complexes^24^has often been used for retrospective evaluation of docking methods and also for evaluation of the classical AC algorithm^21,50^. It contains 18 complexes where the ligand is bound close ($\le$ 5 Å) to a metal ion, most often a zinc ion, but also a heme iron (3 complexes), magnesium (2 cases), or calcium (1 case).

The covalent set CSKDE56 is based on our previously developed CSKDE95 set^46^, filtered additionally for ligands having a molecular electron density score for individual atoms (EDIAm)^51^ above 0.4 and not presenting any crystal contacts with a copy of the receptor at a distance below 4.5 Å, as we found these properties to have a significant influence on the docking predictions^21,46^. The resulting benchmark set consists of 56 high-quality experimental structures (Supporting Information, Table S1), containing 34, 13, 6, 2, and 1 complexes with reactive Cys, Ser, Lys, Glu and Asp residues, respectively. Covalent bonds are formed by 8 distinct chemical reactions, and diverse proteins are present, including 9 protein kinases, 8 cathepsins, and 3 fatty-acid amide hydrolases.

Additionally, we tested our algorithm on a new benchmark set of 70 experimental structures of heme complexes, where a cysteine side chain and a ligand coordinate the heme iron (HemeC70). The same quality filters[46] as for the covalent test set were applied, i.e., keeping only X-ray structures with available electron density, a resolution $\le$ 2.5 Å, and a diffraction-component precision index (DPI)^52^ $\le$ 0.5 Å. The following quality filters were applied to the ligands: average B-factor $\le$ 80 Å^2^, no missing atoms, no alternative conformations, molecular weight in the range [50 Da; 500 Da], less than 18 rotatable bonds, respect at least 3 out of 4 Ghose filters^53^, respect at least 3 out of 4 Lipinski rules^54^, EDIAm score $\ge$ 0.4, and no crystal contacts with a copy of the receptor at a distance below 4.5 Å. This set (Supporting Information, Table S2) contains mainly diverse members of the cytochrome P450 superfamily and of nitric oxide synthases binding to 58 unique ligands, many of them antifungal agents.

All structure and parameter files employed in this study are available on Zenodo (doi: 10.5281/zenodo.15576989).

### QM/MM scheme

**Fig. 1:**
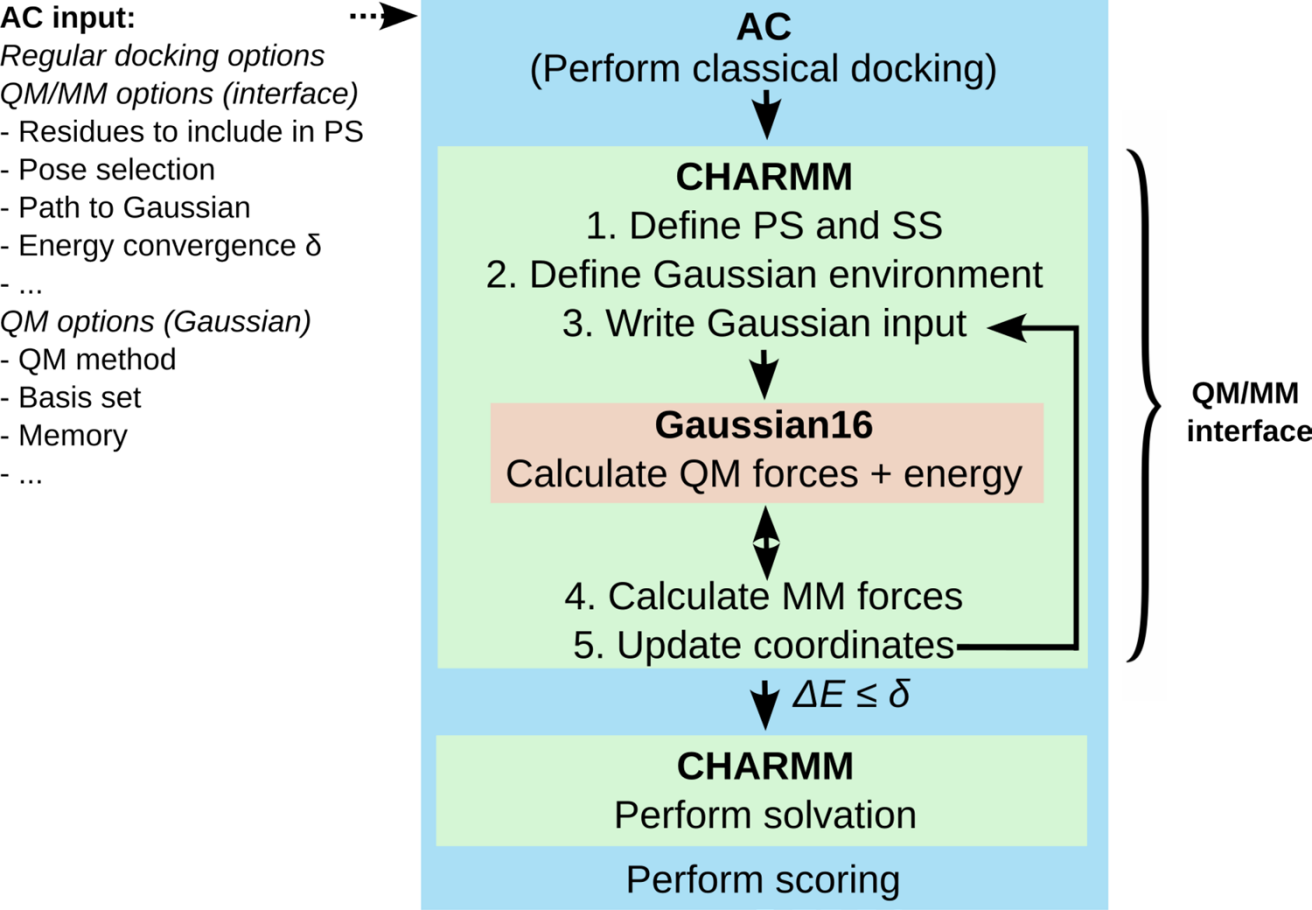
QM/MM procedure in AC. Plain arrows stand for “calls”.

The CHARMM molecular modeling program^23^ contains a QM/MM interface compatible with the Gaussian^49^ quantum mechanics code. In this interface, CHARMM is the main driver and handles dividing the system into a primary system (PS) treated with QM and a secondary system (SS) treated with MM, based on user specifications (Fig. 1). When a covalent bond crosses the PS/SS boundary, a hydrogen link atom is integrated in the PS, and the charge of the first classical neighbor atom is set to zero. The hydrogen link atom, which does not carry any classical point charge nor Lennard-Jones interaction parameters, is aligned to the bond crossing the PS/SS boundary. The interface uses an electrostatic embedding scheme, where the energy and the forces of the PS are calculated at the QM level by Gaussian in presence of the point charges of the SS atoms. Once the QM calculation is completed, the total energy and forces on all atoms in the system are calculated by CHARMM through a subtractive QM/MM scheme (Eq. 1).

1 $$\begin{aligned} \begin{aligned} E_{QMMM}(\text {Complex}) = E_{QM}(\text {PS}) + E_{QM}^{elec}(\text {PS} \leftrightarrow \text {SS}) + E_{MM}(\text {Complex}) - E_{MM}(\text {PS}) - E_{MM}^{elec}(\text {PS} \leftrightarrow \text {SS}) \end{aligned} \end{aligned}$$

The QM energy of the PS, $E_{QM}(\text {PS})$, and the electrostatic interaction term between the PS and the SS, $E_{QM}^{elec}(\text {SS} \leftrightarrow \text {PS})$, are calculated by Gaussian. CHARMM adds the MM energy of the entire system, $E_{MM}(\text {Complex})$, and deduces the MM energy of the PS, $E_{MM}(\text {PS})$. Finally, the MM electrostatic interactions between the PS and the SS, $E_{MM}^{elec}(PS \leftrightarrow SS)$ are excluded since they are already accounted for in the QM energy term $E_{QM}^{elec}(\text {PS} \leftrightarrow \text {SS})$.

If a “non-parametrized” method, such as Hartree-Fock (HF) or DFT, is used to perform the QM calculation, the electrostatic repulsion term among the SS atoms is included in the Gaussian QM energy. It is therefore subtracted a posteriori in the QM/MM scoring procedure of AC to avoid double counting.

### QM/MM docking with AC

The classical AC docking approach^21,46^ consists of successive sampling, optimization, filtering, and scoring steps, which can be controlled by different input options. We implemented the QM/MM docking procedure as additional steps in this process. Poses obtained from classical docking are clustered and filtered before performing a QM/MM optimization and scoring, potentially followed by a refinement optimization and/or scoring at a higher level of QM theory.

In the QM/MM docking approach, three codes communicate as outlined in Fig. 1.

In addition to the parameters for classical docking^21,46,50^, QM/MM docking requires the user to specify the following:

- the atoms to be included in the PS;
- the QM parameters (method, basis set, multiplicity, etc.);
- filtering criteria to limit the number of poses treated by the QM/MM approach (optional);
- a QM/MM energy convergence criterion and/or a maximal number of optimization steps (optional);
- parameters for an additional QM/MM refinement (optional, single-point energy calculation or optimization).

When using multiple CPUs for a classical AC calculation, poses are split among dedicated CPUs^21^. For QM/MM calculations, however, the shared-memory parallelization of Gaussian 16 is used, dedicating all CPUs to the calculation for a single pose.

### Choice of primary system

**Fig. 2:**
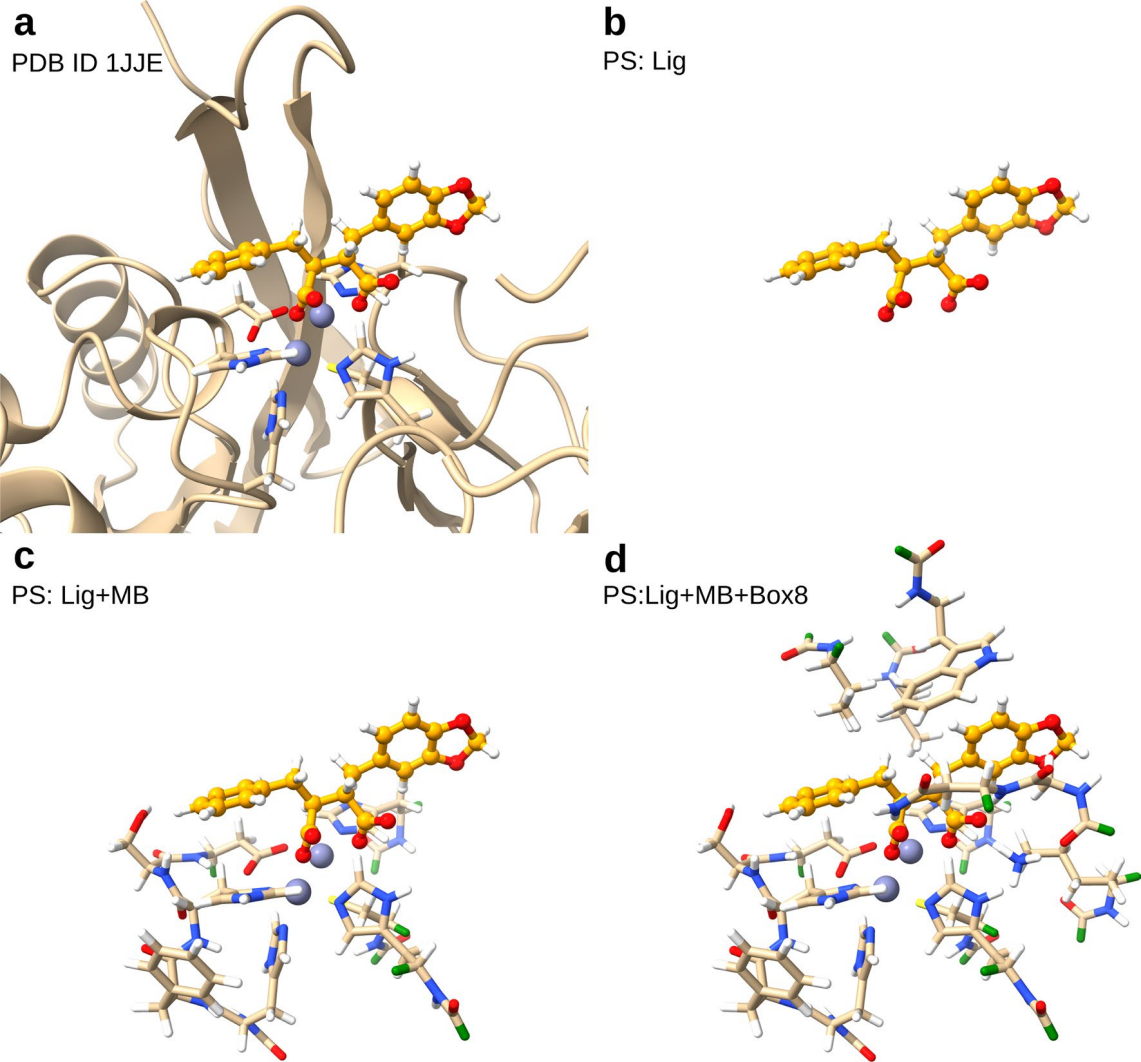
Visualization of different PS employed in this study, shown for a beta-lactamase with a succinic acid inhibitor (PDB ID 1jje). The ligand is shown in orange, the hydrogen link atoms bridging the PS/SS boundary in green. (**a**) Active site structure with inhibitor and two zinc ions, (**b**) PS when only ligand is included. (**c**) PS when ligand, bound metal ions, and coordinating side chains are included. (**d**) PS when additionally a cubic box of 8 Å edge length is defined.

By default, the PS includes the ligand (Lig) and the SS includes the receptor (Rec). In case of covalent docking, the protein binding residue is automatically added to the PS. It is possible to extend the PS by adding specific residues and/or all residues within an orthorhombic box of chosen edge lengths around the center of the sampling box. Additionally, the user can decide whether to include only amino acid side chains or also backbone atoms in the PS. When including only side chains, the boundary is placed at the C$\alpha$–C$\beta$ bond, while it is placed at the C–C$\alpha$ bond when also including backbone atoms. In this work, we tested the following 3 definitions of the PS of increasing size for the QM/MM calculations (Fig. 2):

- only the ligand and, in case of covalent docking, its protein binding residue,
- additionally metal ions in close vicinity of the ligand (distance $\le$ 5 Å) plus their coordinating residues,
- additionally residues within a cubic box of 6/8/10/12 Å edge length around the center of the sampling box.

In the fist case, we included only side chain atoms in the PS, while in the last two cases also backbone atoms were included.

### Scoring and calculation of success rates

In the classical FF-based approach of AC, the scoring function (Score$_{class}$, Eq. (2)) reflects the free energy estimate calculated according to the molecular mechanics–generalized Born surface area (MM-GBSA) method^55^ but neglecting conformational sampling and entropy terms. It can be expressed as:

2 $$\begin{aligned} \begin{aligned} \text {Score}_{class} =&E^{{intra}}(\text {Lig}) + E^{{intra}}(\text {Rec}_{flex}) + E^{{vdW}}(\text {Lig} \leftrightarrow \text {Rec}) + E^{{elec}}(\text {Lig} \leftrightarrow \text {Rec})\\&+ \Delta G^{{solv,elec}}(\text {Complex}) + \Delta G^{{solv,np}}(\text {Complex}) \end{aligned} \end{aligned}$$

Here, the first two terms are the internal energies of the ligand and of the flexible part of the receptor, respectively. The internal energy of the fixed part of the receptor is constant and can be neglected. The third and the fourth terms are the van der Waals and the electrostatic interaction energies between the ligand and the receptor. The last two terms are the polar and non polar solvation energies of the complex, calculated with the fast analytical treatment of solvation (FACTS) model^56^ using a dielectric constant of 2, a nonpolar surface tension coefficient of $0.015 \hbox {kcal/mol/A}^2$and a 12 Å cutoff for nonbonded interactions^21,46,50^. In case of heme-binding ligands, a Morse-like metal binding potential (MMBP) can be added to the scoring function to mimic the energetics of ligand–heme interactions calculated at the DFT level^57^.

The QM/MM score (Eq. 3) was defined accordingly as the sum of the QM/MM energy of the complex (Equation 1) and the FACTS solvation terms:

3 $$\begin{aligned} \begin{aligned} \text {Score}_{QMMM} = E_{\text {QMMM}}(\text {Complex}) + \Delta G^{{solv,elec}}(\text {Complex}) + \Delta G^{{solv,np}}(\text {Complex}) \end{aligned} \end{aligned}$$

To mimic a dielectric constant of 2 and to avoid over-polarization of the QM system by the classical point charges, we divided these charges by 2 during the QM/MM optimization. For the solvation calculation, we tested both the use of the classical point charges for the PS and the use of its Mulliken charges. The final poses are clustered using the same procedure as for classical docking^21,50^.

A re-docking success was defined when the docking pose with the best score had a root-mean-square displacement (RMSD) of $\le$ 1.5 Å with respect to the experimental structure. We chose this cutoff instead of the often employed value of 2 Å to make sure that a successful pose did not significantly deviate from the native pose. A scoring failure was defined when a pose with a better score than the experimental structure and an RMSD above 1.5 Å was found. Symmetry-adapted RMSD values were calculated with the spyrmsd tool^58^ relying on RDKit^59^ for molecule handling.

### Quantum mechanical methods

Gaussian 16^49^ provides access to SE, HF, HF-based, and DFT methods. Here, we tested the two widely used SE methods PM6^60^ and PM7^61^with their default basis set, VSTO-6G (5D, 7F). Both methods are available for more than 80 elements. PM7 is a re-parametrization of PM6 with a focus on the improved treatment of hydrogen bonds^61^. We also carried out DFT refinement with the BYLP functional^62,63^and the B3LYP functional^63–66^, using the split valence polarized (SVP) basis set^67^. Many studies demonstrate the importance of dispersion corrections for biomolecular systems^68–70^. We therefore also carried out calculations using the long-range corrected CAM-B3LYP functional^71^, the larger TZVP basis set, and Grimme’s D3 version of empirical dispersion^72^.

For QM/MM optimizations, we used an energy convergence criterion of 0.05 kcal/mol for the Astex set and of 0.01 kcal/mol for the CSKDS56 and the HemeC70 sets. Restricted closed-shell singlet states were considered in all calculations except for the ones including ferric heme, where an unrestricted open-shell doublet state was considered.

### Sampling parameters

In this study, the QM/MM docking systematically followed classical docking, scoring and clustering procedures. In order to ensure comparability of the results we first carried out classical docking with good sampling parameters determined from previous studies^21,46,50^. For all sets, docking was started from a randomized ligand conformation. A threshold value N$_{Thr}$ of 70 and a ligand rotational angle of 60° were used. For the Astex set, 6 random initial conditions (RIC) and a cubic sampling box with an edge length of 20 Å were used. For the covalent and the heme sets, 4 RIC and a cubic box with an edge length of 25 Å were used to provide a better sampling of larger ligands. The relaxed native pose was then added to the ensemble of poses to ensure its presence in the QM/MM docking runs, except for calculations marked as “on-the-fly”, where QM/MM docking directly followed classical docking and therefore sampling failures can be present. It should be noted that parallel PM7 calculations with Gaussian 16 Revision C.01 are non-deterministic due to numerical errors. This effect can lead to significantly different energies for calculations starting from the same conformation. However, all solutions are meaningful.

### Comparison to other docking codes

We compared the AC docking results to AutoDock Vina version 1.2.3^73,74^ for the Astex and the HemeC70 set, and to AutoDock 4.2^28^ and GOLD^39^ for the Astex and CSKDE56 set. The AutoDock and GOLD calculations were taken from our previous works^19,46^ with the parameters given there. Success rates are reported for docking runs starting from the native ligand pose. AutoDock Vina calculations were carried out here, starting from a randomized ligand conformation, using an exhaustiveness of 100 and a cubic search space of edge length 20 Å (Astex) and 25 Å (HemeC70). In a separate calculation, the native pose was optimized and its score calculated with the *local_only* keyword. The pose with the best score of both calculations was then used to calculate the success rates.

## Results and discussion

### Redocking of non-covalent ligands

We used the Astex Diverse set to test the influence of different parameters on the QM/MM docking approach, as we have manually curated parameters for this set of relevant drug-like ligand–protein complexes. As described above, by default all QM/MM dockings were performed on a classical ensemble of poses including the relaxed native poseand treating the best pose of the best 10 clusters, including the relaxed native pose, in the QM/MM calculations.

The classical scoring function yielded a success rate of 85% (72/85 complexes, Table 1) with an RMSD cutoff of 1.5 Å for the whole set of 85 complexes. For the subset of 18 metal-binding ligands, the success rate was 67%. These success rates demonstrate that the classical scoring function performs very well for non-covalent complexes, but not as successfully for metal-bound ligands. For this set, AC outperforms AutoDock Vina (65%), GOLD with the ChemScore scoring function (67%), and AD (53%).

In QM/MM docking, when including only the ligand in the PS and using the semi-empirical PM7 method, the success rate was 59% for the whole set and 33% for the metal-binding complexes, both much lower than the classical success rate (Table 1). We then decided to include the metal and its coordinating protein side chains in the PS to capture the important electronic effects for the 18 metal-binding complexes. With this approach, the PS contained 61 heavy atoms on average, 132 for metal-binding complexes. Here, the success rate was 65% for the whole set and 61% for the metal-binding complexes, a notable increase with respect to including only the ligand in the PS. The success rate for the metal-binding complexes further increased significantly from 61 to 78% when keeping the metal and its surrounding side chains restrained and optimizing only the ligand pose (Table 1).

In all these calculations, the solvation terms do not significantly increase the success rate, as evident when comparing the success rate (Succ) with the success rate without solvation terms (Succ-ns, Table 1). However, when replacing the FF charges of the PS atoms by QM-derived Mulliken point charges, the success rate increased by 6–10% as compared to the success rates without solvation. When doing an on-the-fly QM/MM docking starting from a randomized ligand conformation, the classical success rate decreased by 7%, the QM/MM success rate by 5%.

We then investigated how the number and the diversity of poses treated by the QM/MM approach influenced the success rates. Up to here, all calculations were done for the best poses of the best 10 clusters, yielding a success rate of 73% with Mulliken point charges and optimizing only the ligand. When including the best poses of only 5 clusters, the success rate increased to 76%, while when including the best pose of 20 clusters, it decreased to 68%. This demonstrates that the success rate decreases with the number of clusters, because there is a higher chance that a non-native pose is scored better than the native pose (scoring failure). However, when including all poses of the first 5 (success rate 81%) or 10 clusters (74%) instead of just the best pose of each cluster, the success rate shows the tendency to increase with the number of poses, because there is a higher chance that a near-native pose with a good score is found. Using the default value of the best poses of the best 10 clusters provides a compromise between computational efficiency and thorough sampling (Table 1).

Testing other QM methods, we observed that PM6 yielded virtually the same results as PM7, contrary to our expectations. For DFT calculations, we did not use the classical poses as input but rather added the calculations in a refinement step after the semi-empirical PM7 optimizations. Adding a single-point DFT calculation with the BLYP functional resulted in 2 complexes with large and charged PS, where the calculations did not converge for any of the 10 poses, and a success rate of 76% for the remaining cases. Single-point DFT calculations with the B3LYP functional yielded slightly better results with no convergence failure and a success rate of 78%. Adding a short optimization at the DFT level using the BLYP functional yielded 10 convergence failures, resulting from complexes with metal-binding or large and charged ligands, and a success rate of 81% for the remaining cases.

We also tested how the docking predictions were influenced by extending the PS, allowing for a better description of direct ligand–target interactions. When including all residues and co-factors with at least one atom inside a cubic box with the same center as the sampling box and edge lengths of 6, 8, 10, and 12 Å, the PS contained on average 100/173/275/422 atoms and 4/8/14/23 residues, respectively. Using these PS definitions and the Mulliken charges for solvation, success rates of 78/73/66/62% were obtained when optimizing all atoms of the PS, and of 79/72/74/75% when optimizing only the ligand. This shows that especially for larger PS, optimizing only the ligand and constraining the rest of the PS is favorable. As also observed in case of classical flexible docking, optimizing many degrees of freedom induces noise in the scoring function and leads to a decreased predictivity. Surprisingly, the smallest PS box size of (6 Å)$^3$ yielded the highest success rate, probably because only including a few crucial residues in the PS.

Investigation of the application of DFT calculations to these extended systems was conducted with a PS defined by a cubic box with an edge length of 8 Å. Addition of a single-point calculation with the BLYP functional resulted in 14 convergence failures and a drastically decreased success rate of 41% for the remaining complexes, suggesting that this functional is ill suited to treat non-covalently bound biomolecular systems. Using the long-range corrected CAM-B3LYP functional with dispersion correction instead, only one (metal-binding) complex did not converge, and the success rate increased to 82% for the whole set, 76% for metal-binding complexes.

In summary, for the Astex Diverse set of standard drug-like non-covalent complexes, using QM-derived point charges for the PS in the solvation calculation and including bound metal ions in the PS led to significant improvements of the QM/MM score. Treatment of the best poses of the best 10 classical clusters is not sufficient to ensure converged success rates, as the inclusion of more diverse poses and of more poses of the same clusters alter the success rate. Extension of the PS to including amino acids and cofactors in the active site can be beneficial. However, manual curation of important residues is likely to outperform the automatic procedure employed in our calculations. The semi-empirical methods PM6 and PM7 show similar results, while DFT approaches including dispersion such as CAM-B3LYP clearly outperform calculations with BLYP and B3LYP functionals for extended systems.

It should be noted that the QM/MM approach is very robust, especially with the semi-empirical methods, leading to very few convergence failures. For metal-binding complexes, both semi-empirical and DFT calculations outperform the classical scoring function, while on the overall test set the classical FF score outperforms all tested QM methods. The CPU time is moderate for SE calculations. Using 4 recent CPU cores, the calculations required about 20 min for 10 poses of the ligand and metal-binding groups, 45 min for all poses of the best 10 clusters, and 20/30/55/70 min for PS box sizes of 6/8/10/12 Å, respectively. The reported DFT calculations required about 70 min for the sp-B3LYP dockings.

**Table:** Docking results for Astex Diverse Set. Success rate (highest scored pose RMSD $\le$ 1.5 Å, Succ); success rate without solvation terms (Succ-ns); fraction of converged cases (Conv); average number of atoms in the PS (#PSAt); average number of poses treated (#Poses). Abbreviations of parameters: bound metals included in PS (MB); cubic box of edge length N Å in PS (BoxN); FF-charges/QM-derived charges for solvation (FF-ch/QM-ch); optimizing PS/ligand only (opt-ps/opt-lig); treating best poses of best N clusters (Npos); treating all poses of best N clusters (Ncl); single-point refinement with specified functional (sp); refinement optimization with specified functional (opt); on-the-fly docking without adding native pose (on-the-fly). If not otherwise stated, PM7 is used for the PS and the best poses of the best 10 clusters (10pos) are treated. Success rates are calculated considering only converged cases.

Parameters	Full set					Metal-binding complexes	
	Succ [%]	Succ-ns [%]	Conv [%]	#PSAt	#Poses	Succ [%]	Conv [%]
Classical	85	71			10	67	100
Classical, on-the-fly	78	66				67	100
Lig, FF-ch	59	60	100	42	10	33	100
Lig+MB, FF-ch, opt-ps	65	62	100	61	10	61	100
Lig+MB, FF-ch, opt-lig	68	66	100	61	10	78	100
Lig+MB, QM-ch, opt-ps	72	62	100	61	10	72	100
Lig+MB, QM-ch, opt-lig	73	66	100	61	10	78	100
Lig+MB, QM-ch, opt-lig, on-the-fly	68	66	100	61	10	56	100
Lig+MB, QM-ch, opt-lig, 5pos	76	68	100	61	5	72	100
Lig+MB, QM-ch, opt-lig, 20pos	68	62	100	61	20	72	100
Lig+MB, QM-ch, opt-lig, 5cl	81	76	100	61	36	72	100
Lig+MB, QM-ch, opt-lig, 10cl	74	72	100	61	68	56	100
Lig+MB, QM-ch, opt-lig, PM6	74	67	100	61	10	78	100
Lig+MB, QM-ch, opt-ps, sp-BLYP	76	66	98	60	10	76	94
Lig+MB, QM-ch, opt-ps, sp-B3LYP	78	66	100	61	10	72	100
Lig+MB, QM-ch, opt-ps, opt-BLYP	81	71	88	54	10	90	56
Lig+Box6+MB, QM-ch, opt-ps	78	71	100	100	10	67	100
Lig+Box8+MB, QM-ch, opt-ps	73	67	100	173	10	72	100
Lig+Box10+MB, QM-ch, opt-ps	66	64	100	275	10	50	100
Lig+Box12+MB, QM-ch, opt-ps	62	61	100	422	10	72	100
Lig+Box6+MB, QM-ch, opt-lig	79	71	100	100	10	78	100
Lig+Box8+MB, QM-ch, opt-lig	72	65	100	173	10	67	100
Lig+Box10+MB, QM-ch, opt-lig	74	66	100	275	10	72	100
Lig+Box12+MB, QM-ch, opt-lig	75	69	100	422	10	72	100
Lig+Box8+MB, QM-ch, opt-lig, sp-BLYP	41	37	84	173	10	27	83
Lig+Box8+MB, QM-ch, opt-lig, sp-CAM-B3LYP	82	71	99	173	10	76	94
AutoDock Vina	65				10	50	100
GOLD, ChemScore^19^	67					61	100
AutoDock^19^	53					33	100

### Redocking of covalent ligands

We tested the redocking of covalent ligands on the CSKDE56 set of 56 diverse high-quality complexes. Following the same procedure as outlined above for non-covalent ligands, i.e. choosing the best poses of the best 10 clusters including the relaxed native pose, the classical scoring function yielded a success rate of 71% (40/56 complexes) with an RMSD cutoff of 1.5 Å (Table 2). GOLD with the PLP scoring function^75^ performed similarly (70%), while AD yielded a lower success rate (49%), suggesting a challenging benchmark set.

For QM/MM docking, the PS contained 48 atoms on average when including only the ligand and its covalently bound protein sidechain. Using the PM7 level of theory, approximately the same success rate as in the classical docking (70%) was obtained regardless of the use of FF or Mulliken charges for the solvation calculation. For on-the-fly docking, both the classical and the QM/MM success rates remained virtually unchanged, suggesting that there are no sampling but just scoring failures.

Adding a single-point DFT calculation with the BLYP functional after the PM7 optimizations did not induce any convergence failures but slightly lowered the success rate to 66%, as well as when using the B3LYP functional (68%). Adding a short DFT optimization with the BLYP/B3LYP functionals again yielded no convergence failures but similar success rates of 68/70%.

When including all residues and co-factors with at least one atom inside a cubic box with the same center as the sampling box and edge lengths of 6, 8, 10, and 12 Å, the PS contained on average 93/158/250/366 atoms and 3/7/13/20 residues, respectively. The success rates were 64/63/64/71%, respectively, not showing a clear trend as a function of the box size and not better than when including only the ligand and the covalently bound side chain in the PS.

**Table 2:** Docking results for covalent CSKDE56 set. For definitions and abbreviations see Table 1. Reactive protein side chain included in PS (SC).

Parameters	Succ [%]	Succ-ns [%]	Conv [%]	#PSAt
Classical	71	66		
Classical, on-the-fly	70	57		
Lig+SC, QM-ch	70	63	100	48
Lig+SC, QM-ch, on-the-fly	71	61	100	48
Lig+SC, QM-ch, PM6	68	63	100	48
Lig+SC, FF-ch	70	63	100	48
Lig+SC, QM-ch, sp-BLYP	66	66	100	48
Lig+SC, QM-ch, sp-B3LYP	68	66	100	48
Lig+SC, QM-ch, sp-CAM-B3LYP	66	64	100	48
Lig+SC, QM-ch, opt-BLYP	68	64	100	48
Lig+SC, QM-ch, opt-B3LYP	70	68	100	48
Lig+SC+Box6, QM-ch, opt-lig	64	61	100	93
Lig+SC+Box8, QM-ch, opt-lig	63	61	100	158
Lig+SC+Box10, QM-ch, opt-lig	64	50	100	250
Lig+SC+Box12, QM-ch, opt-lig	71	57	100	366
GOLD, PLP^46^	70			
AutoDock^46^	49			

**Figure:**
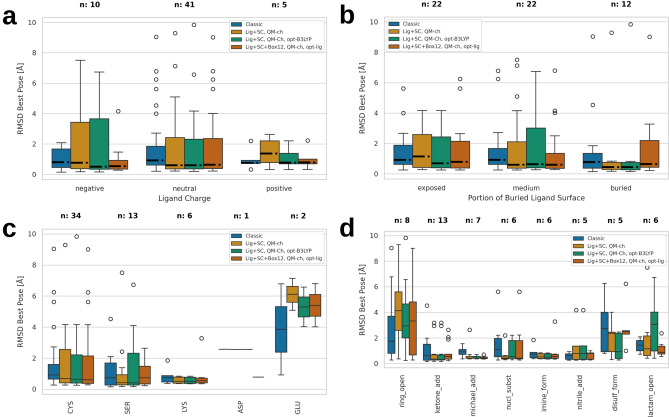
Analysis of classical and QM/MM covalent docking results. Best pose RMSD as a function of (**a**) ligand charge; (**b**) portion of buried ligand surface (exposed, < 0.85; medium, 0.85–0.95; buried, > 0.95); (**c**) covalent protein side chain; and (**d**) chemical reaction type.

Comparison of classical docking results to their QM/MM counterparts (Fig. 3), suggests that generally, difficult cases for QM/MM docking are also difficult for classical docking, e.g., complexes with a covalent bond to a glutamate side chain (Fig. 3c). For charged ligands, especially negatively charged ones, it is beneficial to include the ligand environment in the PS (Fig. 3a), while for very buried ligands the opposite is true (Fig. 3b). B3LYP calculations struggle with the scoring of $\beta$-lactam opening reactions but outperform PM7 for disulfide formation reactions.

**Fig. 4:**
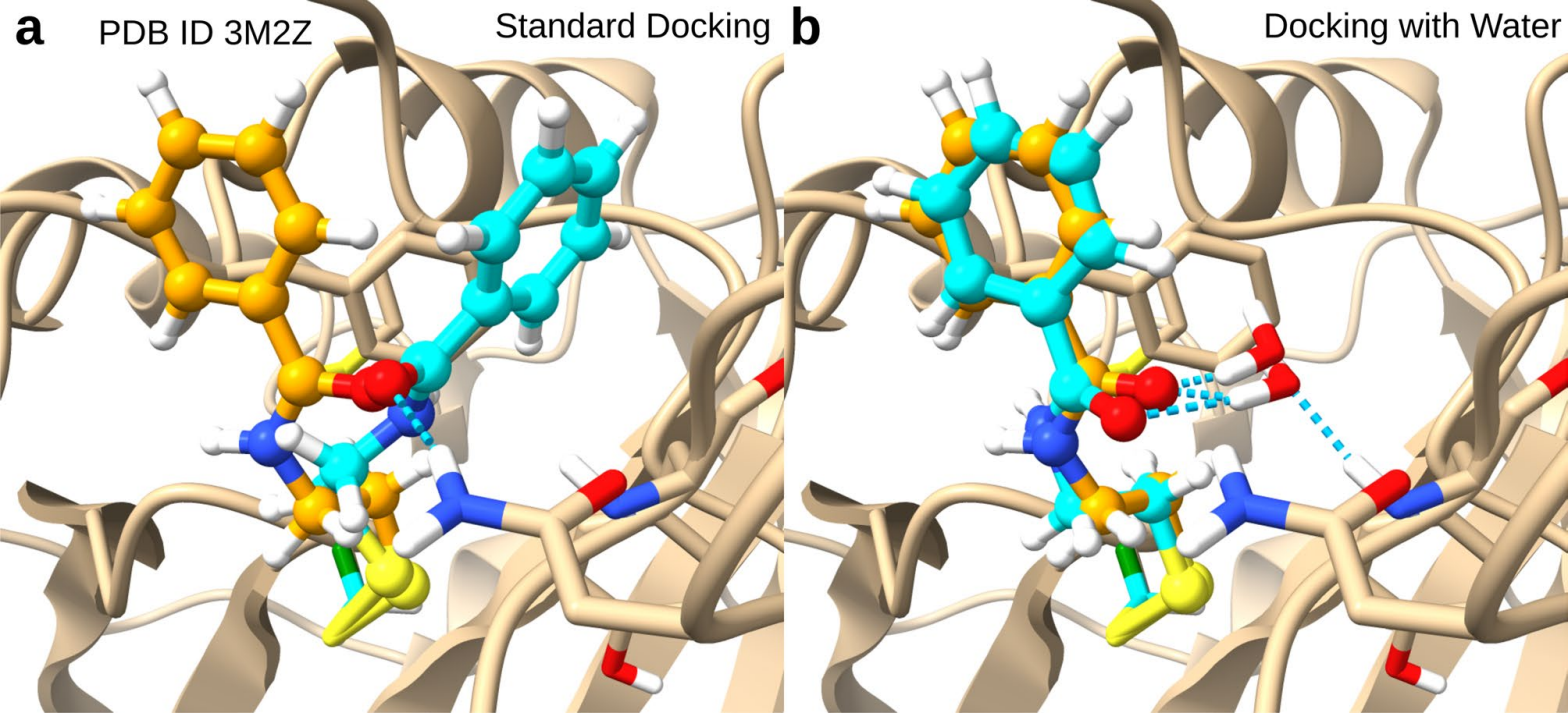
QM/MM docking results of a covalent fragment binding to carbonic anhydrase II H64C mutant (PDB ID 3m2z). The native ligand pose is shown in orange, the best docked poses in cyan, the hydrogen link atom bridging the PS/SS boundary in green. (**a**) Standard docking. (**b**) Docking with one crystallographic water molecule in the PS.

One of the challenges for the QM/MM approach may be missing water interactions. To test this hypothesis, we carried out docking of the N-(2-sulfanylethyl)benzamide fragment to the carbonic anhydrase II H64C mutant (PDB ID 3m2z, Fig. 4) in presence of a water molecule, which is resolved in the structure and shown to form a hydrogen bond with the ligand. Inclusion of this water molecule either in the PS or in the SS reverted the docking failure (best pose RMSD 4.1 Å) to a docking success (RMSD 0.4 and 0.5 Å, respectively, Fig. 4). This finding suggests that a better treatment of solvation and explicit water molecules could indeed yield better docking results.

Contrary to our expectations, the QM/MM approach yields similar results as the classical score for covalently bound ligands. Extension of the PS to surrounding residues or application of a higher level of theory does not seem to improve the results. The discrepancy of these results with previously reported covalent QM/MM docking results using Cov_DOX^42^ cannot be further investigated as we do not have access to the program. CPU time requirements are similar to the non-covalent docking calculations due to similar PS sizes.

### Redocking of heme-binding ligands

To assess the performance of the QM/MM docking algorithm on a more challenging set, we used the high-quality heme-bound ligand complexes of the HemeC70 set. The heme cofactor plays an important role in many physiological redox processes. Its central iron ion exists in two oxidation states, a ferrous reduced Fe(II) state, and a ferric oxidized Fe(III) state. Moreover, depending on its environment, both states can assume different spin states^76^. For simplicity, we only consider low-spin states here, namely a singlet state for ferrous heme and a doublet state for ferric heme.

For the HemeC70 set, the classical AC scoring function reached a success rate of only 16% when considering the ferrous heme state and 40% for the ferric state. Application of MMBPs^57^ to improve the description of ligand–heme interactions led to a success rate of 81% considering ferrous heme and 84% for ferric heme. However, for on-the-fly docking with MMBPs, the success rate was only 60% because of numerous sampling failures, as the ligands are generally quite large and flexible. AutoDock Vina yielded a success rate of 24% on these complexes (Table 3).

In this set, the ligands contain 36 heavy atoms on average, but the PS was significantly bigger with 114 non-hydrogen atoms, because the heme cofactor as well as the iron-coordinating cysteine side chain were always included to capture important electronic interactions. When optimizing only the ligand coordinates at the PM7 level and using Mulliken charges for solvation, a success rate of 80% was obtained for the ferrous complexes, 86% for the ferric complexes. As also seen for classical docking, the on-the-fly QM/MM success rate for ferrous complexes was substantially lower (56%), suggesting that a better sampling is necessary to reach higher success rates. For these complexes, the PM6 method performs much worse, with a success rate of 56% for the ferrous complexes and 64% for the ferric complexes. Also a single-point DFT calculation with the BLYP functional led to a substantially lower success rate (56%) and four convergence failures for the ferrous complexes. DFT calculations for the ferric complexes were too slow to converge and are therefore not reported.

**Fig. 5:**
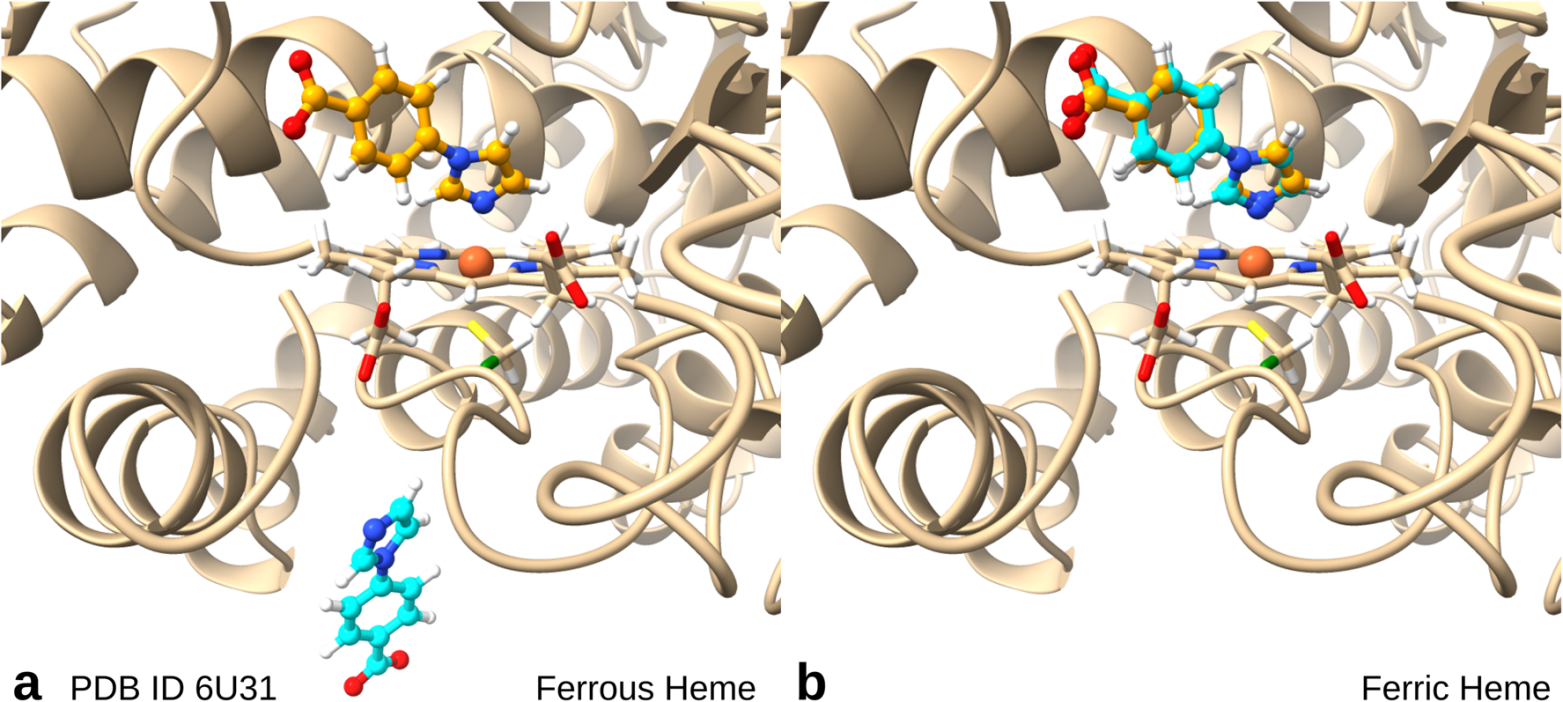
QM/MM docking results of an imidazole ligand to a cytochrome P450 hemeprotein (PDB ID 6u31). The native ligand pose is show in orange, the best docked poses in cyan, the hydrogen link atom bridging the PS/SS boundary in green. (**a**) Docking with ferrous, reduced heme (Fe(II)). (**b**) Docking with ferric, oxidized heme (Fe(III)).

Additional insight can be obtained by the QM treatment. For example, it was shown experimentally that an imidazole ligand of CYP199A4 binds only to ferric heme, but is released upon heme reduction^77^. In agreement with these experimental findings, AC yielded as most favorable pose one with an RMSD of 0.4 Å to the native pose for the ferric complex but one with an RMSD of 17.7 Å for the ferrous complex (Fig. 5). This accuracy cannot be reached with the MMBP approach.

In summary, semiempirical QM/MM docking with the PM7 method yields very high success rates for heme-binding ligands, outperforming the classical scoring function and the PM6 method, and providing physical insight into ligand binding. DFT calculations are difficult to converge on this test set, probably due to the large size of the PS and the highly charged systems. CPU timings for these dockings are somewhat longer than for the other test sets due to the larger number of atoms and electrons in the PS. Docking with PM7 took about 50 min on 4 cores for the ferrous systems and 90 min for the ferric systems.

**Table 3:** Docking results for heme complex set HemeC70. For definitions and abbreviations see Table 1. Heme and coordinating Cys side chain included in PS (HC).

Parameters	Succ [%]	Succ-ns [%]	Conv [%]	#PSAt
Classical ferrous	16	7		
Classical+MMBP ferrous	81	69		
Classical+MMBP, ferrous, on-the-fly	60	46		
Classical ferric	40	49		
Classical+MMBP ferric	84	73		
Lig+HC, QM-ch, opt-lig, ferrous	80	80	100	114
Lig+HC, QM-ch, opt-lig, ferrous, on-the-fly (MMBP)	56	49	100	114
Lig+HC, QM-ch, opt-lig, ferric	86	81	100	114
Lig+HC, QM-ch, opt-lig, ferrous, PM6	56	51	100	114
Lig+HC, QM-ch, opt-lig, ferric, PM6	64	57	100	114
Lig+HC, QM-ch, opt-lig, ferrous, sp-BLYP	56	50	94	114
AutoDock Vina	24			

## Conclusions

In this study we describe a novel QM/MM interface in the docking code Attracting Cavities, extending our previous work^18,19^ to allow docking of non-covalent and covalent ligands at different levels of theory. We assessed the approach on three diverse high-quality benchmark sets of non-covalent, covalent, and heme-binding complexes.

Using QM-derived point charges for the PS and including bound metal ions in the PS is important for obtaining reliable results. Depending on the target and on the task at hand, the number and the nature of poses to be treated at the QM/MM level should carefully be chosen to ensure a sufficient diversity and sufficient sampling of low-energy conformations. Extension of the PS to include amino acids and cofactors in the active site can be beneficial and will be most valuable when done by manual selection of important residues. This should be done for prospective applications, where the user should use prior knowledge and active site visualization to determine which parts of the receptor to include in the PS.

The semi-empirical methods PM6 and PM7 show similar results for non-covalent and covalent complexes, but PM7 largely outperforms PM6 for heme-binding ligands, probably due to its re-parameterization for transition metals^61^. When applying DFT methods, inclusion of dispersion corrections is important, especially for extended PS including many non-covalent interactions.

Classical force-field methods perform very well for non-covalent complexes without metal interactions, for which they have been developed and validated. Additionally, the atomistic structures deposited in the Protein Data Bank, which we use as ground truth, have also been modeled using classical force fields. A QM/MM approach may be expected to work better in the PS but may have deficiencies in describing the PS/SS interactions, boundary effects, and solvation. Their competitive advantage is therefore lowest for non-covalent complexes of drug-like ligands where classical force fields excel, such as the Astex set. We already saw this in our previous semi-empirical QM/MM docking approach^19^.

For covalent complexes, the re-docking success rate of our QM/MM approach on the CSKDE56 set fell short of our expectations raised by previously published work. The underlying reasons are difficult to establish. However, we noted that the QM/MM method is very sensitive to the quality of structural data and prone to failure in cases where atoms are missing or the structure is of bad quality, while FF-based methods are more permissive with respect to such flaws. Inclusion of key water residues may improve docking success. In case of convergence issues or docking failures, different strategies may be pursued, such as the use of better basis sets or a 3-layered QM/MM approach such as ONIOM^78^ instead of the 2-layered approach investigated here.

In conclusion, our QM/MM docking algorithm is very robust, especially with the semi-empirical methods, being applicable to very diverse systems of varying size and leading to very few failures and convergence issues. It outperforms the classical AC code for metalloproteins, reaches similar success rates for covalent complexes, and slightly lower ones for non-covalent complexes. For metal-binding complexes, its improved accuracy justifies its application despite higher CPU time, given the difficulties to accurately describe protein-drug interactions in such systems. However, due to its sensitivity to structural accuracy and CPU requirements, the QM/MM approach is best suited for detailed investigations and small to medium-sized screens. We are confident that its use is beneficial in cases where classical force fields are less reliable, as this study has shown for heme metalloproteins. In the future, we will make this tool freely accessible through a new version of the SwissDock Web server^79^ and assess its usefulness in prospective drug design studies.

## Supplementary Information

Supplementary Information.

## Data Availability

The data used to generate the results of this manuscript (parameter files, topology files, input files, and output analysis scripts) are available on Zenodo (https://doi.org/10.5281/zenodo.15576989). The docking code will be made available through the SwissDock Web server (www.swissdock.ch) in the near future.
