# Supplementary material for: Hybrid quantum/classical docking of covalent and non-covalent ligands with Attracting Cavities
Source: Sci Rep. 2025 Nov 26;15:42271. doi: 10.1038/s41598-025-24614-3 (PMC12657515; doi:10.1038/s41598-025-24614-3)
Supplement: Supplementary file 1 — Supplementary Information. [file 41598_2025_24614_MOESM1_ESM.pdf]

# Supporting Information: Hybrid Quantum/Classical Docking of Covalent and Non-Covalent Ligands with Attracting Cavities

**Mathilde Goullieux**[1,+], **Vincent Zoete**[1,2], **and Ute F. Röhrig**[1,*]

[1]SIB Swiss Institute of Bioinformatics, Molecular Modeling Group, CH-1015 Lausanne, Switzerland

[2]Department of Oncology UNIL-CHUV, Lausanne University, Ludwig Institute for Cancer Research Lausanne Branch, CH-1066 Epalinges

[+]Current address: Center For Electron Laser Science CFEL, Deutsches Elektronen-Synchrotron DESY, Notkestr. 85, 22607 Hamburg, Germany

[*]ute.roehrig@sib.swiss

## Contents

## List of Tables

S1 Covalent complex set . . . . . . . . . . . . . . . . . . . . . . . . . . . . . . . . . . . . . . . . . . . 3

S2 Heme complex set . . . . . . . . . . . . . . . . . . . . . . . . . . . . . . . . . . . . . . . . . . . . 5

## Supporting Tables

| Case | LigID | EDIAm | Burial | #DOF | Charge | ProtSite | Reaction |
|---|---|---|---|---|---|---|---|
| 1fj8 | CER | 0.68 | 0.97 | 8 | 0 | Cys163 | ring_open |
| 1hbj | FBQ | 0.90 | 0.99 | 6 | 0 | Ser200 | ketone_add |
| 1qdq | 074 | 0.86 | 0.88 | 13 | -1 | Cys29 | ring_open |
| 1td2 | PXL | 0.77 | 0.97 | 2 | 0 | Cys122 | aldehyde_add |
| 2awz | 5H | 0.74 | 0.88 | 5 | 0 | Cys366 | michael_add |
| 2dw5 | BFB | 0.71 | 0.75 | 9 | 1 | Cys645 | nucl_subst |
| 2g8e | 0M6 | 0.78 | 0.75 | 12 | 0 | Cys115 | ketone_add |
| 2op9 | WR1 | 0.56 | 0.79 | 13 | 0 | Cys145 | ring_open |
| 2wj1 | S99 | 0.97 | 0.98 | 9 | 0 | Ser241 | ketone_add |
| 2z9w | PXL | 0.94 | 0.98 | 2 | 0 | Lys197 | imine_form |
| 3h0e | H0E | 0.68 | 0.76 | 5 | 0 | Cys163 | ketone_add |
| 3hhi | 074 | 1.00 | 0.81 | 13 | -1 | Cys122 | ring_open |
| 3k7f | F2C | 0.98 | 0.98 | 10 | -1 | Ser241 | ketone_add |
| 3kwb | ORH | 0.81 | 0.72 | 6 | 0 | Cys1025 | nitrile_add |
| 3m2z | BFE | 0.88 | 0.80 | 4 | 0 | Cys64 | disulf_form |
| 3mzd | CXV | 0.65 | 0.84 | 7 | 0 | Ser44 | b_lactam_open |
| 3oj8 | OJ8 | 1.01 | 0.98 | 5 | 0 | Ser241 | ketone_add |
| 3ovx | O64 | 0.54 | 0.78 | 6 | 0 | Cys25 | ketone_add |
| 3pdf | LXV | 0.96 | 0.87 | 4 | 0 | Cys234 | nitrile_add |
| 3q7z | BOU | 0.97 | 0.74 | 8 | -2 | Ser389 | b_lactam_open |
| 3svv | VSP | 0.95 | 0.94 | 6 | 0 | Cys338 | michael_add |
| 3t9t | IAQ | 0.97 | 0.85 | 7 | 1 | Cys442 | michael_add |
| 3zmh | L62 | 0.94 | 1.00 | 7 | 0 | Ser201 | b_lactam_open |
| 4amz | 2P4 | 0.99 | 0.84 | 8 | 0 | Ser554 | ketone_add |
| 4an1 | 2P8 | 0.96 | 0.83 | 8 | 0 | Ser554 | ketone_add |
| 4cdc | 6AO | 0.82 | 0.79 | 8 | 1 | Cys234 | nitrile_add |
| 4cde | U6B | 0.79 | 0.73 | 7 | 1 | Cys234 | nitrile_add |
| 4cdf | W2C | 0.92 | 0.75 | 7 | 1 | Cys234 | nitrile_add |
| 4cl8 | OZJ | 0.87 | 0.90 | 2 | 0 | Cys168 | aldehyde_add |
| 4jxg | 1S6 | 0.83 | 0.85 | 7 | -1 | Ser64 | b_lactam_open |
| 4lqm | DJK | 0.74 | 0.88 | 5 | 0 | Cys797 | michael_add |
| 4luc | 20G | 0.83 | 0.94 | 9 | 0 | Cys12 | disulf_form |
| 4mll | 1S6 | 0.65 | 0.92 | 7 | -1 | Ser67 | b_lactam_open |
| 4qgv | LMC | 0.90 | 0.95 | 3 | 0 | Lys132 | imine_form |
| 4qps | 37Q | 0.93 | 0.91 | 6 | 0 | Cys909 | michael_add |
| 4wsk | 3U2 | 0.85 | 0.79 | 3 | 0 | Asp229 | ring_open |
| 5ac0 | K9P | 0.77 | 0.84 | 5 | 0 | Cys301 | nucl_subst |
| 5ac2 | K9P | 0.74 | 0.90 | 5 | 0 | Cys302 | nucl_subst |

| | | | | | | | |
|---|---|---|---|---|---|---|---|
| 5d11 | 56G | 0.92 | 0.73 | 8 | 0 | Cys345 | michael_add |
| 5d6e | 94A | 0.89 | 0.86 | 7 | 0 | Glu364 | ring_open |
| 5e1i | 6B8 | 0.50 | 0.90 | 5 | -1 | Cys354 | aldehyde_add |
| 5j9z | 6HJ | 0.88 | 0.91 | 4 | 0 | Cys797 | michael_add |
| 5l6p | 6P8 | 0.88 | 0.87 | 4 | 0 | Cys717 | nucl_subst |
| 5mjb | 7O3 | 0.94 | 0.88 | 5 | 0 | Cys703 | nucl_subst |
| 5orl | A4W | 0.77 | 0.85 | 4 | 0 | Cys247 | disulf_form |
| 5qh8 | H0A | 0.70 | 0.84 | 3 | 0 | Cys73 | nucl_subst |
| 5xhr | PXH | 0.77 | 0.87 | 7 | -1 | Ser64 | b_lactam_open |
| 6aff | 72U | 0.94 | 0.66 | 2 | 0 | Lys148 | imine_form |
| 6ary | BT7 | 0.71 | 0.99 | 5 | 0 | Ser360 | ketone_add |
| 6c7z | JLO | 0.44 | 0.98 | 3 | 0 | Lys108 | imine_form |
| 6eej | J6S | 0.88 | 0.98 | 5 | -1 | Lys122 | imine_form |
| 6hmu | GE8 | 0.64 | 0.81 | 7 | 0 | Cys45 | disulf_form |
| 6hn2 | GF8 | 0.59 | 0.76 | 5 | 0 | Cys42 | disulf_form |
| 6j7b | BJL | 0.90 | 0.92 | 10 | -1 | Cys169 | ring_open |
| 6puh | OYG | 1.04 | 0.99 | 2 | 0 | Lys43 | imine_form |
| 6q6l | RJR | 0.71 | 0.95 | 8 | 0 | Glu340 | ring_open |

**Table S1.** Covalent complex cases: ligand PDB ID (LigID), EDIAm value, ligand burial, ligand number of rotatable bonds (#DOF), ligand charge, covalently bound protein residue (ProtSite), chemical reaction.

| Case | LigID | EDIAm | Burial | #DOF | Charge |
|---|---|---|---|---|---|
| 1p2y | NCT | 0.68 | 1.00 | 2 | 1 |
| 1suo | CPZ | 0.95 | 1.00 | 1 | 0 |
| 2fdu | D1G | 0.73 | 1.00 | 5 | 0 |
| 2fdv | D2G | 0.97 | 1.00 | 4 | 0 |
| 2fdw | D3G | 0.81 | 1.00 | 3 | 0 |
| 2oro | 228 | 0.98 | 0.76 | 6 | 0 |
| 2w0b | CMW | 0.80 | 0.98 | 8 | 0 |
| 2wuz | TPF | 0.68 | 0.96 | 6 | 0 |
| 2wx2 | TPF | 0.76 | 0.98 | 6 | 0 |
| 3a51 | VDY | 0.58 | 0.91 | 12 | 0 |
| 3b6h | MXD | 0.88 | 0.99 | 1 | 0 |
| 3ibd | CPZ | 0.74 | 1.00 | 1 | 0 |
| 3mdm | FJZ | 0.93 | 1.00 | 2 | 0 |
| 3mdr | GJZ | 0.83 | 0.98 | 2 | 0 |
| 3qoa | 3QO | 0.66 | 1.00 | 2 | 0 |
| 3swz | TOK | 0.63 | 1.00 | 4 | 0 |
| 3t3q | 9PL | 0.58 | 1.00 | 5 | 0 |
| 3t3r | 9PL | 0.47 | 1.00 | 5 | 0 |
| 3t3z | 9PL | 0.61 | 1.00 | 5 | 0 |
| 4d32 | 7F5 | 0.55 | 0.85 | 8 | 1 |
| 4d33 | 6J0 | 0.62 | 0.87 | 6 | 1 |
| 4d34 | E2Z | 0.49 | 0.80 | 6 | 1 |
| 4d38 | L5Z | 0.55 | 0.81 | 6 | 1 |
| 4d39 | OLW | 0.73 | 0.89 | 5 | 1 |
| 4d3b | 6J0 | 0.60 | 0.86 | 6 | 1 |
| 4dtw | SRO | 0.72 | 0.95 | 4 | 0 |
| 4dtz | LDP | 0.94 | 0.97 | 5 | 0 |
| 4du2 | LDP | 0.88 | 0.99 | 5 | 0 |
| 4dub | LDP | 0.99 | 0.97 | 5 | 0 |
| 4due | SRO | 0.90 | 0.98 | 4 | 0 |
| 4duf | SRO | 0.41 | 0.97 | 4 | 0 |
| 4eji | 0QA | 0.78 | 1.00 | 7 | 0 |
| 4g44 | TZM | 0.85 | 0.89 | 3 | 0 |
| 4g45 | MQN | 0.89 | 0.88 | 2 | 0 |
| 4tt5 | 36Y | 0.86 | 0.98 | 1 | 0 |
| 4uch | OLW | 0.39 | 0.86 | 5 | 1 |
| 4v3u | EG8 | 0.63 | 0.84 | 7 | 1 |
| 4v3w | E2Z | 0.56 | 0.83 | 6 | 1 |
| 4v3x | 4E8 | 0.78 | 0.87 | 7 | 1 |
| 4v3y | HLW | 0.72 | 0.84 | 7 | 1 |
| 4v3z | 9HL | 0.53 | 0.82 | 6 | 1 |

| | | | | | |
|---|---|---|---|---|---|
| 4wmz | TPF | 0.96 | 0.99 | 6 | 0 |
| 4wpd | 3SQ | 0.89 | 0.99 | 1 | 0 |
| 4xrz | SI6 | 0.62 | 0.97 | 2 | 1 |
| 4zdz | TPF | 0.84 | 0.99 | 6 | 0 |
| 4ze0 | VOR | 0.78 | 0.99 | 7 | 0 |
| 5ead | 5L9 | 0.95 | 0.99 | 6 | 0 |
| 5eae | 5L8 | 0.90 | 1.00 | 6 | 0 |
| 5ese | TPF | 0.85 | 0.99 | 6 | 0 |
| 5esf | TPF | 0.75 | 0.99 | 6 | 0 |
| 5esj | TPF | 0.87 | 0.99 | 6 | 0 |
| 5esm | TPF | 1.03 | 0.99 | 6 | 0 |
| 5hs1 | VOR | 0.94 | 1.00 | 7 | 0 |
| 5ibd | GGJ | 0.88 | 0.94 | 7 | 0 |
| 5ibe | 69M | 0.83 | 0.95 | 6 | 0 |
| 5ibf | 69W | 0.82 | 0.96 | 4 | 0 |
| 5ibg | 69S | 0.79 | 0.91 | 7 | 0 |
| 5l92 | C0R | 0.65 | 0.91 | 6 | 0 |
| 5li7 | 6XD | 0.94 | 0.85 | 5 | 0 |
| 5o4k | 9KE | 0.57 | 0.94 | 6 | 0 |
| 5o4l | 9KB | 0.56 | 0.94 | 6 | 0 |
| 6a16 | UCZ | 0.91 | 1.00 | 4 | 0 |
| 6csd | PN0 | 0.61 | 1.00 | 8 | 0 |
| 6h1l | FJQ | 0.90 | 0.95 | 6 | 0 |
| 6h1o | VOR | 0.90 | 0.98 | 7 | 0 |
| 6h1s | TPF | 0.85 | 0.98 | 6 | 0 |
| 6h1t | CL6 | 0.80 | 0.96 | 4 | 0 |
| 6ma7 | TPF | 0.85 | 0.92 | 6 | 0 |
| 6u30 | PQM | 0.97 | 1.00 | 2 | -1 |
| 6u31 | PQP | 0.81 | 1.00 | 2 | -1 |

**Table S2.** Heme complex cases: ligand PDB ID (LigID), EDIAm value, ligand burial, ligand number of rotatable bonds (#DOF), ligand charge.
